# Gadoterate meglumine decreases ADC values of breast lesions depending on the b value combination

**DOI:** 10.1038/s41598-017-18035-0

**Published:** 2018-01-08

**Authors:** Otso Arponen, Mazen Sudah, Anna Sutela, Mikko Taina, Amro Masarwah, Timo Liimatainen, Ritva Vanninen

**Affiliations:** 1 Kuopio University Hospital, Diagnostic Imaging Centre, Department of Clinical Radiology, Kuopio University Hospital, PO Box 100, Puijonlaaksontie 2, 70029 Kuopio, Finland; 2 University of Eastern Finland, Institute of Clinical Medicine, School of Medicine, Department of Clinical Radiology, Kuopio University Hospital, PO Box 1777, Puijonlaaksontie 2, 70210 Kuopio, Finland; 3 University of Eastern Finland, Cancer Center of Eastern Finland, Yliopistonranta 1, 70210 Kuopio, Finland

## Abstract

To retrospectively evaluated the influence of administration of the gadolinium based intravenous contrast agent (G-CA) on apparent diffusion coefficient (ADC) values in ADC maps generated using multiple b value combinations. A total of 106 women underwent bilateral 3.0 T breast MRI. As an internal validation, diffusion-weighted imaging (b values of 0, 200, 400, 600, 800 s/mm^2^) was performed before and after the G-CA (gadoterate meglumine (0.2 ml/kg, 3 ml/s)). Whole lesion and fibroglandular tissue (FGT) covering region-of-interests (ROIs) were drawn on the b = 800 s/mm^2^ images; ROIs were then propagated to multiple retrospectively generated ADC maps. Twenty-seven patients (mean age 55.8 ± 10.8 years) with 32 mass-like enhancing breast lesions including 25 (78.1 %) histopathologically malignant lesions were enrolled. Lesion ADC values were statistically significantly higher in pre-G-CA than post-G-CA ADC maps (ADC_0,200,400,600,800_: 1.05 ± 0.35 × 10^−3^ mm^2^/s vs. 1.02 ± 0.36 × 10^−3^ mm^2^/s (*P* < 0.05); ADC_0,200,400_: 1.25 ± 0.42 × 10^−3^ mm^2^/s vs. 1.20 ± 0.35 × 10^−3^ mm^2^/s (*P* < 0.05)). ADC values between pre- and post-contrast maps were not statistically different when the maps were generated using other b value combinations. Contrast agent administration did not affect the FGT ADC values. G-CA statistically significantly reduced the ADC values of breast lesions on ADC maps generated using the clinically widely utilized b values.

## Introduction

Breast MRI has emerged as the most sensitive imaging technique in the detection and evaluation of breast lesions; it has proved to be an important adjunct to mammography in the preoperative local staging of breast cancer and has been suggested to benefit women in average to high breast cancer risk populations as a screening method. The morphological and kinetic features of MRI achieve excellent sensitivity (87 % to 99 %) although with somewhat lower specificity (81 % to 89 %) in the characterization of breast lesions^1,2^.

The inclusion of diffusion-weighted imaging (DWI) in the breast MRI protocols is a promising technique which is a subject of on-going research^3^. The diffusion reflected in the values of the apparent diffusion coefficient (ADC) indirectly describes the random (Brownian) motion of the water molecules in living tissues^4^. The movement of water within different compartments (intracellular, extracellular and extravascular, and intravascular), as well as the molecular exchange between compartments affect the ADC values^5,6^. In breast lesions, ADC values are affected by tissue cellularity, fluid viscosity, membrane permeability, macromolecular structures, microvascularity and tumor blood flow^7–10^. Addition of DWI in conjunction with routine MRI sequences has been demonstrated to increase the specificity of the BI-RADS guideline assisted breast lesion interpretation^11–14^. Despite promising results in clinical practice^3^, the overlap of ADC values between malignant and benign lesions not only in different reports but even within the same study, has hindered the establishment of universal ADC thresholds and optimal imaging protocol^15^.

Gadolinium based intravenous contrast agents (G-CA) are routinely used when performing breast MRI, however their effects on ADC values of breast lesions remain uncertain and the published results are inconsistent^16–19^. Decreases in breast lesion ADC values due to gadolinium based intravenous contrast agent (G-CA) have been reported to be as substantial as −11 % to −23 % when using 1.5 T scanners^17,18^. In contrast, when using a 3.0 T scanner, Nguyen *et al*. reported no significant change in ADC values after the G-CA^19^. Yuen *et al*. speculated that the change in ADC values with regard to G-CA could be caused by a microperfusion effect^17^, while other investigators have proposed that contrast medium eliminates pseudo-diffusion contributions^17,20^ or causes local magnetic field susceptibilities^21–23^.

The possible relationship between gadolinium-based contrast agents and the different b value combinations on DWI has not been determined. In this study, we aimed to compare the ADC values obtained from breast lesions and fibroglandular tissue (FGT) before and after dynamic contrast enhanced (DCE) MRI using ADC maps generated using numerous combinations from two to five clinically relevant b values.

## Materials and Methods

### Study Design and Patients

Patients with suspicious breast findings are referred to our tertiary hospital (catchment area 260,000 inhabitants) for consultation and further management. Between August 2015 and January 2016, 106 women who met the European Society of Breast Cancer Specialists working group (EUSOMA) guideline criteria underwent bilateral 3.0 T breast MRI. As an internal validation, DW imaging (b values of 0, 200, 400, 600, 800 s/mm^2^) was performed before and immediately after DCE sequences (360 seconds after the G-CA injection). The study was approved by the Kuopio University Hospital Research Ethics Board and all clinical investigations have been conducted according to the relevant guidelines and the principles expressed in the Declaration of Helsinki. The need for written informed consent was waived by the local chair of the Kuopio University Hospital district.

A total of 42 consecutive women with 48 mass-enhancing lesions that were DWI-visible and subsequently histopathologically confirmed were primarily included in this retrospective analysis. Patients were excluded if the exact localization of the breast lesion could not be precisely assessed or did not remain identical on each ADC map due to one of the following reasons: 1) patient/breast movement between the two DWI-scans, 2) poor or inaccurate demarcation of the lesion dimensions on the DWI map compared to DCE/T2w images, or 3) small lesions (< 30 pixels) on the ADC maps. Measurement of the lesion ADC values was considered to be unreliable in 16 lesions due to breast movement between sequences (N = 1), poor lesion demarcation on DWI map (N = 10) or small lesion size (N = 5). Therefore, the final study sample meeting the inclusion criteria consisted of 27 women with 32 breast lesions. Non-mass like enhancing lesions were excluded.

Breast MRIs were primarily evaluated by breast radiologists with 20 years of experience in breast radiology. The findings were managed according to the BI-RADS guideline recommendations^3^. For the purposes of the present study, pre- and post-contrast ADC maps were retrospectively generated on a pixel-by-pixel manner from DW images with varying b values. The DWI validation protocol did not change the diagnostic decisions or the management of the patients.

### Breast MRI Protocol

MRI examinations were performed in the prone position with a 7-element phased-array coil dedicated to bilateral breast imaging (Philips Achieva 3.0 T TX, Philips N.V., Eindhoven, The Netherlands). The structural breast MRI protocol consisted of five sequences. The T1-weighted fast field echo sequence (TR = 4.58 ms; TE (in phase) = 2.3 ms; in-plane resolution 0.48 mm × 0.48 mm; 257 slices; slice thickness 0.7 mm) was followed by a T2-weighted turbo spin echo sequence (TR = 5000 ms; TE = 120 ms, flip angle 90°; in-plane resolution 0.6 mm × 0.6 mm; 85 slices; slice thickness 2 mm) and a short T1-inversion recovery/turbo spin echo (TR = 5000 ms; TE = 60 ms; TI 230 ms; in-plane resolution 1 mm × 1 mm; 90 slices; slice thickness 2 mm). A contrast-enhanced dynamic eTHRIVE sequence (TR = 4.66 ms; TE = 2.3 ms; spectrally adiabatic inversion recovery (SPAIR) fat suppression; dynamic scan time 58.5 s; in-plane resolution 0.96 mm × 0.96 mm; 180 slices; slice thickness 1 mm with pre-contrast and six phases after the G-CA. Contrast agent (gadoterate meglumine (0.2 ml/kg, 3 ml/s)) was injected intravenously followed by a bolus of saline chaser. DWI echo planar imaging (TR (shortest) = 6982 ms (range 6982–7789 ms); TE = 95 ms; flip angle 90°; SPAIR fat suppression; in-plane resolution 1.15 mm × 1.15 mm; 30 slices; slice thickness 4 mm; diffusion gradients in four directions) with five respective b factors (0, 200, 400, 600, and 800 s/mm^2^) was performed before and after the G-CA. The b values were selected to achieve the optimal clinical performance at 3.0 T^19,24^.

### Generation of pre- and post-contrast ADC maps with different b value combinations and the ROI placement

Identical imaging parameters were used for pre- and post-contrast DWI sequences. Post-contrast DWI was performed 360 seconds after the injection of the contrast agent. Pre- and post-contrast ADC maps were generated (O.A) on a pixel-by-pixel manner from diffusion-weighted images with varying b values by fitting a mono-exponential function from an open-source toolkit AEDES (aedes.uef.fi) running on the Matlab platform (The Mathworks, Natick, MA).

**Figure 1:**
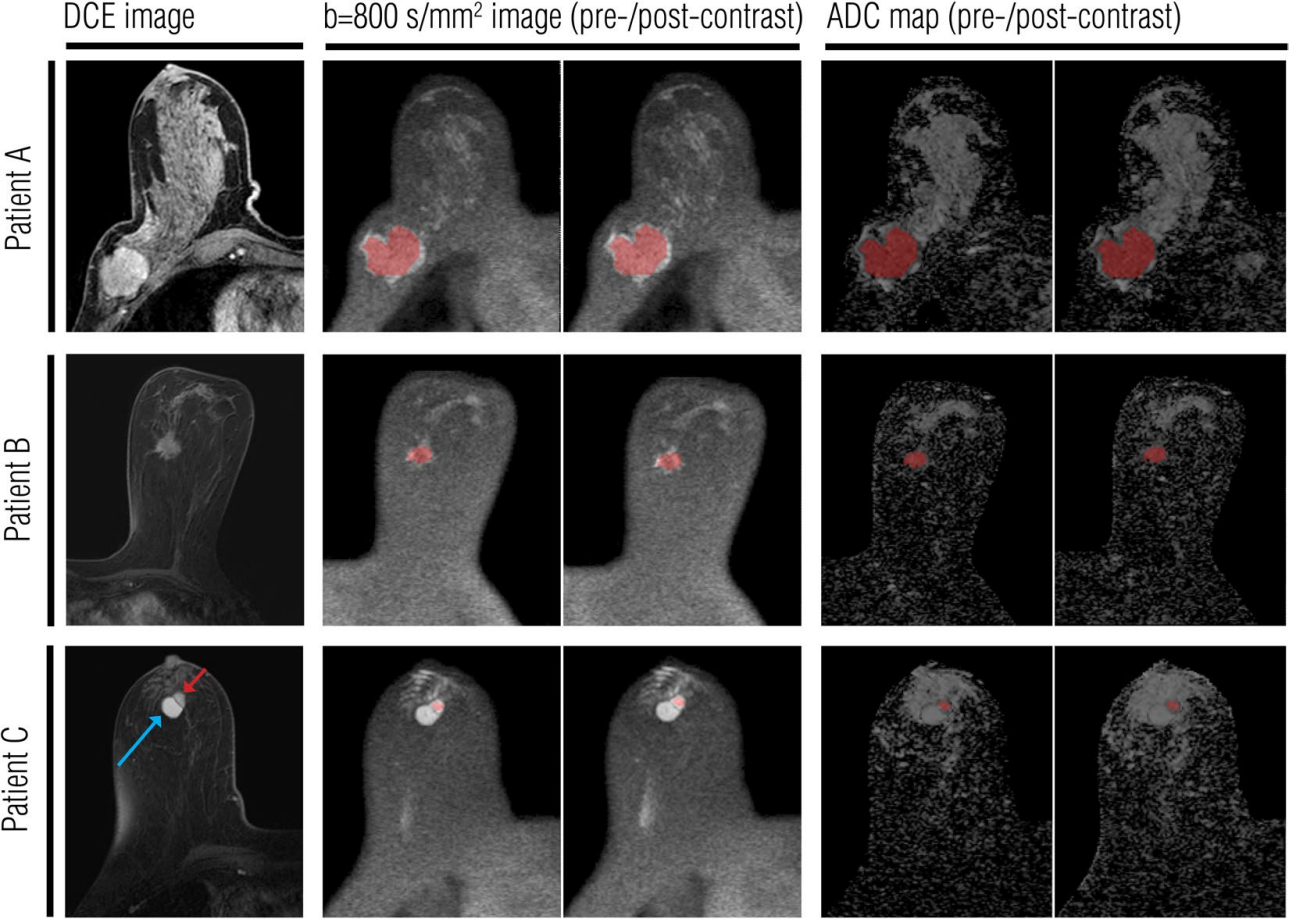
Pre- and post-contrast ADC measurements in three illustrative lesions. The lesions were first localized using T1-weighted, T2-weighted and dynamic contrast enhanced (DCE) images (DCE images shown in the left column). A whole lesion covering region of interest (ROI) was drawn on the pre-contrast b = 800 s/mm^2^ image. This ROI was then replicated on pre- and post-contrast ADC maps. Images in the right column show ROIs on the pre- and post-contrast ADC maps that were generated using the b values of 0, 200, 400, 600, and 800 s/mm^2^. (**A**) A large ductal carcinoma of 47 mm in diameter in the right breast. (**B**) A ductolobular carcinoma of 21 mm in diameter in the left breast. (**C**) An intraductal papilloma (red arrow) of 13 mm in diameter in the right breast. A circumscribed, oval hemorrhagic cyst (blue arrow) was detected lateral to the enhancing mass.

Using the crosshair tool (Sectra PACS, version 162 15.1.20.2, Sectra Workstation IDS7, Linköping, Sweden), T1-weighted, T2-weighted and DCE images were used to localize the lesions and the FGT on b = 800 s/mm^2^ DW images (Fig. 1). Carefully avoiding cystic, necrotic and fatty areas, a whole lesion covering ROI and a FGT covering ROI were drawn on b = 800 s/mm^2^ images on AEDES; if possible, the same ROI was used to measure both the pre- and post-G-CA ADC values. The localization of the ROI was adjusted to compensate for the possible slight movement of the breast between examinations. Lesion and FGT ROIs were propagated to other ADC maps generated using different b value combinations. The percentage change for ADC values in each tissue type (lesion, FGT) was calculated as follows: (ADC_post-G-CA_ − ADC_pre-G-CA_)/ADC_pre-G-CA_ × 100 %. Contrast-to-noise ratios (CNRs) were calculated according to Yuen *et al*., using the following equation: CNR = (SI_lesion_ − SI_fat_)/[(SI_lesion_ + SI_fat_)/2], where SI refers to the signal intensity for lesions and fat in each b value combination^17^.

### Histopathological evaluation of the lesions

Histopathological samples were obtained using 14-gauge core needles and targeted ultrasound. Core biopsy (CB) specimens were placed into 10 % formalin and embedded in paraffin after fixation. The samples were cut into 5-µm slices at four different levels and stained with haematoxylin and eosin (HE). In the case of carcinomas, the final diagnosis was reconfirmed from surgical specimens.

### Statistical Analysis

Continuous variables are presented as mean ± standard deviation (SD) and categorical variables as absolute values and percentages. The normality of the distribution was evaluated with the Kolmogorov-Smirnov and Saphiro-Wilk tests. Pre- and post-contrast ADC measurements were compared separately for lesions and FGT using the paired Student’s t-test and the Wilcoxon signed-rank test for normally and non-normally distributed variables, respectively. Spearman rank-order correlation between pre- and post-contrast ADC measurements was assessed separately for lesions and for FGT. The comparison between ADC values of the malignant and benign lesions was performed with Mann-Whitney U test. Statistical significance was set at *P* < 0.05. Data was analyzed using IBM SPSS Statistics for Macintosh, Version 22.0.

## Results

Twenty-seven women (mean age 55.8 ± 10.8 years (range 39.2–73.4 years)) with 32 mass lesions were subdivided as follows; 24 (75.0 %) invasive carcinomas, 1 (3.1 %) non-invasive carcinoma (DCIS) and 7 (21.9 %) benign lesions). The mean maximal diameter of the lesions was 2.0 ± 1.1 cm (range 0.9–5.5 cm) being 1.4 ± 0.4 cm (range 0.9–2.0 cm) for the benign lesions and 2.2 ± 1.2 cm (range 0.9–5.5 cm) for the malignant lesions. Detailed descriptions of the histopathological subtype, size and a set of ADC measurements for each lesion are presented in the supplemental material (Supplemental materials, Table 1).

**Table 1:** Tissue type and mean ± standard deviation apparent diffusion coefficient (ADC) values by using different b value combinations before and after the gadolinium based contrast agent administration (G-CA).

b Value Protocol	Tissue type							
	Lesion (N = 32)				Fibroglandular tissue (N = 32)			
	Pre-G-CA ADC (x 10^−3^ mm^2^/s)	Post-G-CA ADC (x 10^−3^ mm^2^/s)	Mean change (%)	r/*P*	Pre-G-CA ADC (x 10^−3^ mm^2^/s)	Post-G-CA ADC (x 10^−3^ mm^2^/s)	Mean change (%)	r/*P*
ADC_0,200,400,600,800_	1.05 ± 0.35	1.03 ± 0.36	−1.58 ± 4.55	0.984/0.042	1.49 ± 0.28	1.48 ± 0.28	−0.42 ± 4.47	0.950/ns.
ADC_400,600,800_	0.87 ± 0.30	0.88 ± 0.36	0.87 ± 13.7	0.930/ns.	1.25 ± 0.31	1.28 ± 0.30	2.22 ± 12.23	0.876/ns.
ADC_0,200,400_	1.25 ± 0.42	1.20 ± 0.35	−2.15 ± 8.07	0.968/0.016	1.73 ± 0.26	1.70 ± 0.26	−1.51 ± 4.81	0.956/ns.
ADC_0,200,800_	1.03 ± 0.35	1.02 ± 0.35	−1.55 ± 4.86	0.980/ns.	1.46 ± 0.28	1.46 ± 0.28	−0.24 ± 4.53	0.956/ns.
ADC_0,400,800_	1.06 ± 0.35	1.04 ± 0.35	−1.46 ± 4.91	0.979/ns.	1.49 ± 0.28	1.48 ± 0.27	−0.24 ± 4.43	0.958/ns.
ADC_0,200_	1.38 ± 0.45	1.35 ± 0.44	−0.70 ± 8.93	0.951/ns.	1.86 ± 0.26	1.86 ± 0.27	−0.01 ± 7.08	0.910/ns.
ADC_0,800_	1.06 ± 0.35	1.04 ± 0.35	−1.46 ± 4.90	0.979/ns.	1.49 ± 0.28	1.48 ± 0.27	−0.24 ± 4.43	0.955/ns.
ADC_200,800_	0.83 ± 0.28	0.80 ± 0.33	−2.53 ± 23.1	0.902/ns.	1.17 ± 0.30	1.20 ± 0.30	3.72 ± 9.84	0.895/0.05

ADC = Apparent Diffusion Coefficient; G-CA = gadolinium based intravenous contrast agent; Pre-G-CA = measurement before the intravenously administered contrast agent; Post-G-CA = measurement after the intravenously administered contrast agent.

**Figure 2:**
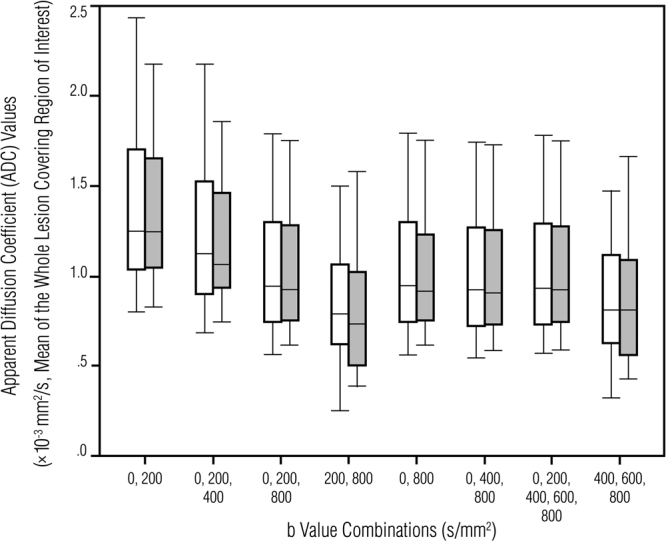
Box-and-whiskers plots showing pre-contrast (white boxes) and post-contrast (gray boxes) breast lesion ADC values generated using different b value combinations. The boxes represent the interquartile range (the data from the 25th to the 75th percentile); the horizontal line in the box refers to the median ADC value and the whiskers show the range (minimum and maximum) of the mean ADC values.

The mean ADC values of the lesions varied according to the b value combination (Fig. 2). In the malignant breast lesions, mean ADC values ranged between 0.83–1.38 × 10^−3^ mm^2^/s for pre-G-CA and 0.80–1.35 × 10^−3^ mm^2^/s for post-G-CA maps (Table 2). In the benign breast lesions, mean ADC values ranged between 1.17–1.87 × 10^−3^ mm^2^/s for pre-G-CA and 1.19–1.78 × 10^−3^ mm^2^/s for post-G-CA maps (Table 2). The ADC values were significantly lower in malignant versus benign lesions (all b value combinations *P* < 0.01). There was a strong correlation in ADC values of breast lesions (r = 0.902–0.984) and FGT (r = 0.876–0.958) in pre- and post-contrast measurements (Table 1). Post-contrast ADC values were significantly lower than the pre-contrast ADC values in all lesions, when all b values (ADC_0,200,400,600,800_) and low b values (ADC_0,200,400_) were used to generate the ADC maps. The mean reductions in post-contrast ADC values were 1.58 % for ADC_0,200,400,600,800_ (*P* = 0.042) and 2.15 % for ADC_0,200,400_ (*P* = 0.016). In the ADC_0,200,400,600,800_ maps, the reduction caused by the G-CA remained significant when only malignant lesions were included (1.60 %, *P* = 0.042). In contrast, when b values of 0 and 200 (ADC_0,200_) were used, no significant change was observed. In FGT, G-CA did not statistically change the ADC values, however, in ADC_200,800_ the mean change was 3.72 % (*P* = 0.05). A detailed analysis of changes in ADC values generated by different b value combinations is presented in Table 2. Image quality was assessed by CNR measurements; mean ± SD values for pre- and post-contrast images are presented in Table 3. There was no significant difference in image quality between pre- and post-contrast images (Table 3).

**Table 2:** Indicate lesion type and mean ± standard deviation apparent diffusion coefficient (ADC) values by using different b value combinations before and after the gadolinium based contrast agent administration (G-CA).

b Value Protocol	Tissue type							
	Malignant (N = 25)				Benign (N = 7)			
	Pre-G-CA (x 10^−3^ mm^2^/s)	Post-G-CA (x 10^−3^ mm^2^/s)	Mean change (%)	r/*P*	Pre-G-CA (x 10^−3^ mm^2^/s)	Post-G-CA (x 10^−3^ mm^2^/s)	Mean change (%)	r/*P*
ADC_0,200,400,600,800_	0.93 ± 0.28	0.92 ± 0.28	−1.60 ± 4.51	0.975/0.042	1.47 ± 0.25	1.46 ± 0.30	−1.49 ± 5.07	1.00/ns.
ADC_400,600,800_	0.76 ± 0.24	0.76 ± 0.27	0.21 ± 13.3	0.902/ns.	1.25 ± 0.17	1.30 ± 0.33	3.23 ± 15.80	0.857/ns.
ADC_0,200,400_	1.12 ± 0.34	1.08 ± 0.28	−1.43 ± 8.61	0.947/ns.	1.71 ± 0.34	1.62 ± 0.29	−4.73 ± 5.46	0.750/ns.
ADC_0,200,800_	0.92 ± 0.28	0.90 ± 0.27	−1.57 ± 4.64	0.969/0.040	1.45 ± 0.24	1.44 ± 0.30	−1.50 ± 6.00	1.000/ns.
ADC_0,400,800_	0.94 ± 0.28	0.92 ± 0.27	−1.36 ± 4.82	0.970/ns.	1.48 ± 0.25	1.46 ± 0.30	−1.79 ± 5.61	0.964/ns.
ADC_0,200_	1.25 ± 0.37	1.23 ± 0.30	0.40 ± 9.60	0.925/ns.	1.87 ± 0.35	1.78 ± 0.34	−4.62 ± 4.55	0.929/ns.
ADC_0,800_	0.94 ± 0.28	0.92 ± 0.27	−1.36 ± 4.82	0.970/ns.	1.48 ± 0.25	1.46 ± 0.30	−1.79 ± 5.61	0.964/ns.
ADC_200,800_	0.73 ± 0.23	0.69 ± 0.23	−3.32 ± 24.0	0.875/ns.	1.17 ± 0.18	1.19 ± 0.35	0.31 ± 20.8	0.929/ns.

ADC = Apparent Diffusion Coefficient; G-CA = gadolinium based intravenous contrast agent; Pre-G-CA = measurement before the intravenously administered contrast agent; Post-G-CA = measurement after the intravenously administered contrast agent.

**Table 3:** Contrast-to-noise ratio (CNR) means ± standard deviations in images obtained before and after the administration of gadolinium based intravenous contrast agent (G-CA) using fat as the reference value.

b Value Protocol	Pre-G-CA CRN	Post-G-CA CRN	*P* value
ADC_0,200,400,600,800_	0.55 ± 0.07	0.55 ± 0.09	ns.
ADC_400,600,800_	0.64 ± 0.13	0.62 ± 0.13	ns.
ADC_0,200,400_	0.50 ± 0.18	0.51 ± 0.23	ns.
ADC_0,200,800_	0.54 ± 0.07	0.55 ± 0.10	ns.
ADC_0,400,800_	0.54 ± 0.09	0.54 ± 0.12	ns.
ADC_0,200_	0.57 ± 0.53	0.64 ± 0.78	ns.
ADC_0,800_	0.54 ± 0.09	0.54 ± 0.12	ns.
ADC_200,800_	0.54 ± 0.26	0.54 ± 0.24	ns.

Pre-G-CA = measurement before the intravenously administered contrast agent; Post-G-CA = measurement after the intravenously administered contrast agent; CNR = Contrast-to-Noise Ratio; ADC = Apparent Diffusion Coefficient. *P* value indicates the statistical difference between the CNRs in pre- and post-contrast images.

## Discussion

The roles of G-CA, different b value combinations and their interplay affecting the ADC values are controversial and the literature about the subject is scanty (Table 4). Our study reveals that ADC values were significantly lower after G-CA in a sample of 32 breast mass-lesions imaged at 3.0 T. Furthermore, no support could be found for the hypothesis that it would be the microperfusion effect causing the reduction.

**Table 4:** Review of the literature about the effects of contrast agents on apparent diffusion coefficient (ADC) values in breast tumors.

Number of patients (N of lesions)	Rubesova *et al*.^16^	Yuen *et al*.^17^	Janka R *et al*.^18^	Nguyen *et al*. (2016)	Present study
	5 (7; 2 benign, 5 malignant)	19 (19; 19 malignant)	n/a (35; 10 benign, 25 malignant)	19 (19; 19 malignant of which 14 mass-like enhancing and 5 NMLE lesions)	27 (32; 7 benign, 25 malignant, all lesions mass-like enhancing lesions)
MRI field strength, vendor, manufacturer	1.5 T, Magnetom Symphony, Siemens	1.5 T, Gyroscan Intera Nova Dual, Philips	1.5 T, Magnetom Avanto, Siemens	3.0 T, Achieva Tx, Philips	Achieva TX, Philips, Achieva Tx, Philips
b value combinations	n/a	ADC_0,1000_	ADC_50,400,800_	ADC_0,100,800_; ADC_0,800_; ADC_100,800_	ADC_0,200,400,600,800_; ADC_400, 600,800_; ADC_0,200,400_; ADC_0,200,800_; ADC_0,400,800_; ADC_0,200_; ADC_0,800_; ADC_200,800_
Contrast agent (dose mmol/kg)	Gadodiamide (0.3)	Gadopentetate dimeglumine (0.1)	Gadobutrol (0.1)	Gadoteridol (0.1)	Gadoterate meglumine (0.2)
DCE-MRI acquisition time (minutes)	15	8	6	9	6
Median TR/TE (ms)	2600/110	1206/71	4100/98	5336/61	6982/95
Mean ± SD pre-contrast/post-contrast ADC values mm^2^/s (*P*)	n/a (ns.)	1.35 ± 0.38/1.04 ± 0.34 (*P* = 0.01)	Benign lesions: 1.99 ± 0.37/1.97 ± 0.30 (*P* = ns.). Malignant lesions: 0.90 ± 0.14/0.80 ± 0.14 (*P* < 0.01)	ADC_0,100,800_: 1.10/1.10 (*P* = ns.)^a^ ADC_0,800_: 1.14/1.09 (*P* = ns.)^a^ ^A^ ADC_100,800_: 0.92/0.87 (*P* = ns.)^a^	All lesions ADC_0,200,400,600,800_: 1.05 ± 0.35/1.03 ± 0.36 (*P* = 0.042). Other b value combinations: no significant differences in ADC values (*P* = ns.). Malignant lesions: ADC_0,200,400,600,800_: 0.93 ± 0.28/0.92 ± 0.28 (*P* = 0.042); ADC_0,200,400_: 0.92 ± 0.28/0.90 ± 0.27 (*P* = 0.040). Other b value combinations: no significant differences in ADC values (*P* = ns.). Benign lesions: No significant differences in ADC values (*P* = ns.).

N = number; n/a = not applicable information; ns. = not significant; TR = repetition time; TE = echo time; DCE-MRI = dynamic contrast enhanced magnetic resonance imaging.

^a^Instead of mean pre- and post-G-CA ADC values, the authors report median values.

There is no consensus in the recent literature about the role of intravenous gadolinium based contrast agent administration or how different b value combinations and their interplay influence the ADC values (Table 4). Our results are in agreement with the results obtained with a 1.5 T scanner reported by Janka *et al*. (b = 50, 400, 800 s/mm^2^) and Yuen *et al*. (b = 0, 1000 s/mm^2^), who also detected decreased ADC values in breast lesions after contrast agent administration^17,18^. However, the mean decrease of ADC values in their samples was more prominent (−11 % to −23 %, respectively) compared to that detected in our study sample (−2 %) (Tables 2 and 3). In contrast, Rubesova *et al*. in a sample of 7 breast lesions (b values not presented) at 1.5 T and Nguyen *et al*. in a sample of 19 lesions at 3.0 T using three b value combinations (ADC_0,100,800_, ADC_0,800_, ADC_100,800_) did not report statistically significantly reduced breast lesion ADC values after the contrast agent administration which might be attributed to smaller sample sizes and can be partially caused by inclusion of non-mass enhancing lesions (Table 4). Nevertheless, both reported lower post-contrast ADC values (Table 4).

It has been previously suggested that differences in field strengths (3.0 T vs. 1.5 T) and types of contrast agent could explain the discrepancies in the literature^19^. Despite the fact that the ADC values remain similar irrespective of field strength^25^, a higher magnetic field could reduce the contrast-induced effect on ADC values due to the theoretical relative decrease in contrast agent induced shortening of intrinsic T1 and T2. The use of contrast materials that reportedly have lower relaxivities (r1 and r2) could also contribute to the results when the field strengths vary^19,26^. Rohrer *et al*. reported that the T1 relaxivities were 3.7 l mmol^−1^ s^−1^ and 3.5 l mmol^−1^ s^−1^ whereas the corresponding T2 relaxivity values were 5.7 l mmol^−1^ s^−1^ and 4.9 l mmol^−1^ s^−1^ in plasma phantoms with the contrast agents used in Nguyen’s study and in our study (Table 4); it is noteworthy that both relaxivities overlap in accuracy ranges^26^. In addition to differences in field strengths and contrast agents, repetition times (TR) and echo times (TE) used in these studies vary (Table 3). Nguyen *et al*. hypothesized that a short TR would prevent complete longitudinal relaxation between excitation pulses in breast tissue leading to T1 saturation effects and SNR reductions in the DWI signal^19^ which could account for some of the discrepancies. We are not aware of any studies confirming the role of TR and TE in 3.0 T DWI accuracy. However, preclinical studies showed that with 1.5 T devices, the use of TR > 3000 ms and TE ≤ 100 ms did not change the ADC values^27^ but that long TR and short TE were likely to increase the accuracy in ADC quantification^28^.

The motion of the water molecules caused by both diffusion and perfusion has been speculated to contribute to ADC values^29^. Some studies have proposed that the contrast agent affects the ADC values by suppressing the microperfusion effect^17,20,30^. Microperfusion is claimed to increase the ADC values in breast lesions, especially when low b values (< 100–150 s/mm^2^) have been applied to generate ADC maps^6,31^. Although there are some investigators who do not believe that microperfusion makes any actual contribution to ADC values in breast tissue^32,33^, it is widely acknowledged in the literature that the microperfusion effect declines when higher b values are used^4,24^. In our patient sample, the ADC maps generated using low b values that emphasize microperfusion weighting (ADC_0,200_) produced higher ADC values than those generated with clinically applied combinations (Fig. 2). However, in ADC_0,200_, the gadolinium based contrast material did not significantly affect the ADC values, suggesting that microperfusion is not the key factor behind the reduction of the post-contrast ADC values. The difference between the pre- and post-contrast ADC values is therefore attributable to interstitial contrast material sequestration that either eliminates pseudo-diffusion contributions^20^ or causes local magnetic field susceptibilities^21–23^; the magnitude of these effects may be partially explained by different relaxivities (r1 and r2) when the field strengths vary^19,26^.

As previously addressed, multiple factors might influence pre- and post-contrast ADC values; yet the exact mechanism remains uncertain. Although our results show that gadolinium based contrast injection results in decreased ADC values, this decrease is minimal and it can be further speculated that it is of little or even no clinical significance. However, Janka *et al*. noted that post-contrast ADC values were lower only in malignant lesions, not in benign lesion, and suggested that this could be of clinical relevance because it makes the distinction between malignant and benign lesions easier^18^. We are not aware of other publications and therefore more research on the subject is advocated. Although the DCE breast MRI sequence remains the gold standard in the primary characterization of breast lesions^3^, regardless of its many limitations, DWI, has emerged as an invaluable supplement to the traditional sequences. Increased imaging time might increase inconvenience to the patient and thus decrease compliance. Therefore, in our institution, we give priority to the contrast-enhanced dynamic sequence and only perform the DWI-sequences after they have been obtained. This emphasizes the need for breast MRI protocol standardization and local validation of ADC values in each breast center. For research purposes, pre-contrast DW sequences might be advocated to avoid the possible effects of gadolinium based contrast agents.

Our patient sample is rather small, which is a major limitation of our study. Furthermore, only a limited number of different b values were used. Several studies suggest that the choice should be a b value combination of 0 s/mm^2^ with b_max_ in a range between 750–1000 s/mm^2^ at 1.5 T^34^. At 3 T, a b value combination of 50 and 850 s/mm^2^ have been suggested for optimal ADC determination and DW imaging quality^25^. The b values in this study were chosen according to the previous literature and have been used routinely in our prospectively collected institutional database.

To conclude, G-CA significantly reduces the ADC values of breast lesions on ADC maps generated using several clinically utilized b value combinations. The magnitude of this change depends on which b values are being used. The effect of contrast agent should be taken into consideration when comparing different studies. Accordingly, in clinical practice, breast DWI should be performed systematically before or after the administration of the contrast agent.

## Electronic supplementary material

Supplemental materials
