# Supplementary material for: Gadoterate meglumine decreases ADC values of breast lesions depending on the b value combination
Source: Sci Rep. 2018 Jan 8;8:87. doi: 10.1038/s41598-017-18035-0 (PMC5758819; doi:10.1038/s41598-017-18035-0)

**Gadoterate meglumine decreases ADC values of breast lesions depending on the b value combination**

Otso Arponen MD, Mazen Sudah MD PhD, Anna Sutela MD PhD, Mikko Taina MD PhD, Amro Masarwah MD PhD, Timo Liimatainen M.Sc. PhD, Ritva Vanninen MD PhD.


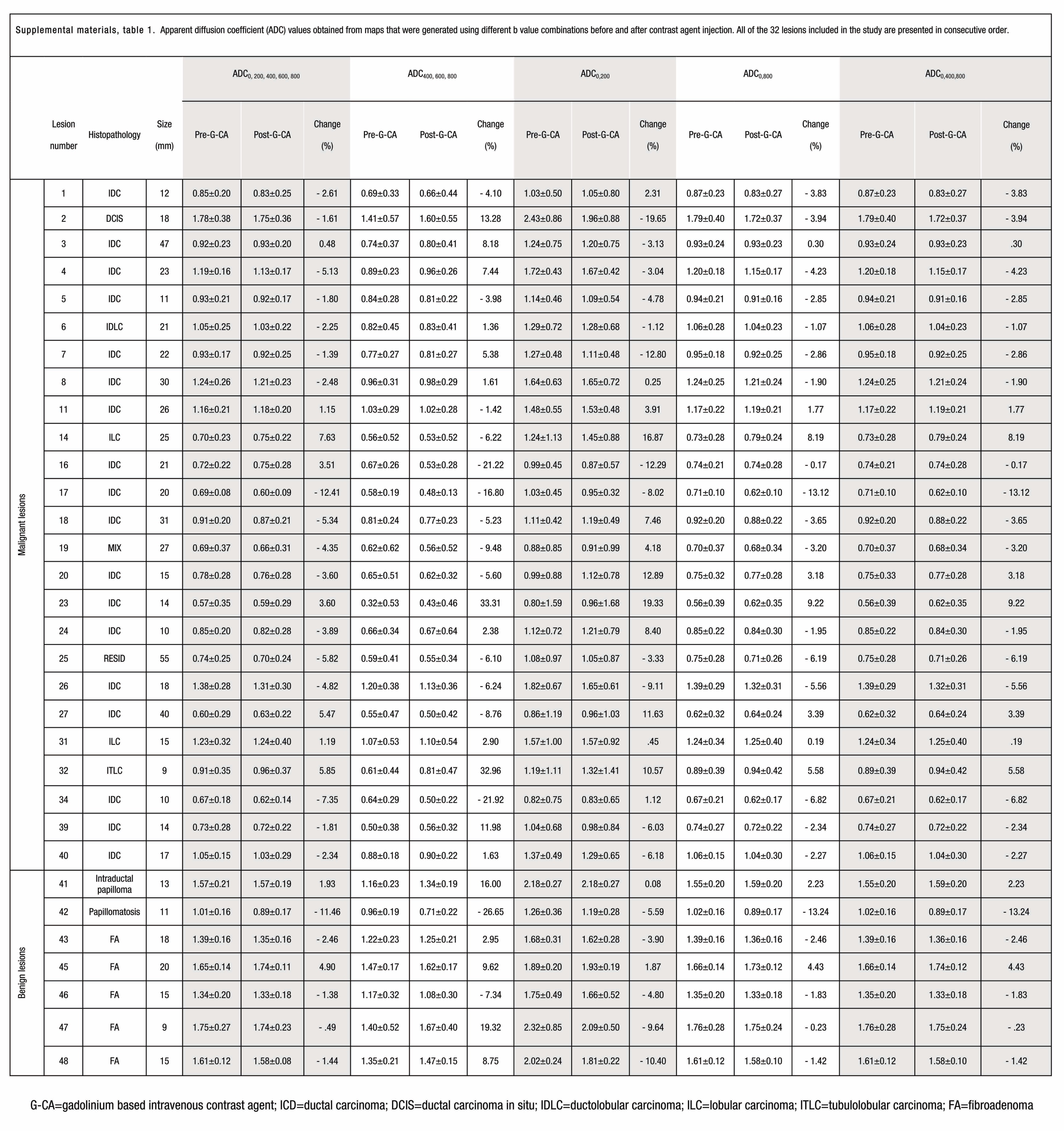

Supplement: Supplementary file 1 — Supplemental materials [file 41598_2017_18035_MOESM1_ESM.doc]
